# Sodium-Glucose Cotransporter 2 Inhibitors Improve Chronic Diabetic Macular Edema

**DOI:** 10.1155/2020/8867079

**Published:** 2020-11-12

**Authors:** Yoko Takatsuna, Ryoichi Ishibashi, Tomoaki Tatsumi, Masaya Koshizaka, Takayuki Baba, Shuichi Yamamoto, Koutaro Yokote

**Affiliations:** ^1^Department of Ophthalmology, Chiba Rosai Hospital, Ichihara, Chiba, Japan; ^2^Department of Ophthalmology and Visual Science, Chiba University Graduate School of Medicine, Chiba, Japan; ^3^Department of Medicine, Division of Diabetes, Endocrinology and Metabolism, Kimitsu Chuo Hospital, Kisarazu, Chiba, Japan; ^4^Department of Endocrinology, Hematology and Gerontology, Graduate School of Medicine, Chiba University, Chiba, Japan; ^5^Division of Diabetes, Metabolism and Endocrinology, Chiba University Hospital, Chiba, Japan

## Abstract

**Purpose:**

Diabetic macular edema (DME) is a vision-threatening condition that develops in diabetic patients. The first-line therapy for DME is intravitreal injections of antivascular endothelial growth factor (anti-VEGF) agents; however, the high frequency of repeat injections, invasiveness of the procedure, and high cost are drawbacks for this treatment. The purpose of this report is to present our findings in 3 patients with chronic DME whose edema was resolved soon after oral doses of sodium-glucose cotransporter-2 (SGLT2) inhibitors were used. *Case Presentation*. Case 1 was a 66-year-old woman diagnosed with moderate nonproliferative diabetic retinopathy (DR) with DME that had developed a decade earlier. The DME persisted for 4 years in the left eye. The addition of oral empagliflozin, a SGLT2 inhibitor, led to a marked improvement of the DME after one month, and this improvement continued over two years. Case 2 was a 68-year-old woman who was diagnosed with preproliferative DR with bilateral DME. The addition of oral dapagliflozin led to the improvement of the DME after two months, and this improvement continued over one year. Case 3 was a 61-year-old woman who was diagnosed with moderate nonproliferative DR with DME. Oral luseogliflozin was given which led to better glycemic control, and her left central retinal thickness (CRT) was markedly reduced after only two weeks. This reduction was maintained in her left eye for six months without any additional ophthalmic procedures.

**Conclusions:**

Although this study involved only three cases, our findings indicate that SGLT2 inhibitors might have possible efficacy for chronic DME.

## 1. Introduction

Diabetic retinopathy (DR) is a frequent cause of blindness in working-age individuals. Diabetic macular edema (DME) is one of the vision-reducing conditions that is associated with DR, and the number of patients with DME increases with the advancement of the retinopathy.

At present, the standard treatment for DME is intravitreal injections of anti-vascular endothelial growth factor (VEGF) agents. A number of clinical studies have shown the superiority of anti-VEGF therapy over laser photocoagulation or steroid injections to treat DME [1, 2]. However, there are some problems with the anti-VEGF therapy; the need for frequent repeat injections and the high cost of anti-VEGF agents are obstacles for their more frequent use. In addition, DME can persist even after the anti-VEGF treatment in approximately 31.6 to 65.6% of the patients [3].

Sodium-glucose cotransporter 2 (SGLT2) inhibitors are a new treatment option for patients with type 2 diabetes mellitus that improves the glycemic control by inhibiting the reabsorption of glucose in the proximal renal tubules leading to an increase in renal glucose extraction [4]. Approximately 90% of the glucose in glomerular filtrates is reabsorbed by the proximal renal tubule and returned to the blood. SGLT2 is present in the proximal renal tubule, and it cotransports glucose and sodium from the glomerular filtrate back into the blood. SGLT2 inhibitors interfere with the glucose reabsorption, and the glucose is excreted in the urine. Recent large-scale clinical trials have demonstrated that SGLT2 inhibitors prevent or reduce mortality, heart failures [5], and the progression of renal impairment [6] in diabetic patients. In addition, studies have shown the presence of SGLT2 in retinal pericytes and mesangial cells [7]. Hyperglycemia results in functional and morphological changes in these cells, but these effects are attenuated by SGLT inhibitors. Although SGLT inhibitors have been shown to be helpful in maintaining the general condition in diabetic patients, their effect on the DME in patients with DR has not been determined.

We report our findings in three patients with type 2 diabetes with chronic DME who were refractory to anti-VEGF therapies and other therapies. However, the three cases had a marked improvement of the DME soon after an oral SGLT2 inhibitor was begun. All patients had chronic DME lasting for more than 5 years and had undergone laser and/or anti-VEGF and/or steroid injections.

## 2. Case Presentation

### 2.1. Case 1

**Figure 1:**
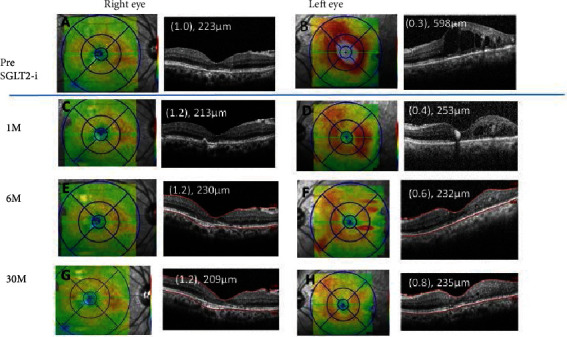
Case 1. Optical coherence tomographic images and fundus photographs showing the central retinal thickness (CRT) pre (A, B), at 1 month (M) (C, D), 6 M (E, F), and 30 M (G, H) after commencing the oral sodium-glucose cotransporter 2 inhibitor (SGLT2i).

A 66-year-old woman was diagnosed with moderate nonproliferative DR with DME bilaterally a decade earlier. She underwent focal laser photocoagulation in both eyes, and the decimal best-corrected visual acuity (BCVA) in her right eye improved to 1.0, but it did not improve in her left eye. She did not agree to further therapy such as anti-VEGF for the left eye, because the BCVA in her right eye was good and the high cost of anti-VEGF agents. The macular edema in the left eye persisted for 4 years with a decimal BCVA of 0.3 and a central retinal thickness (CRT) of 598 *μ*m (Figure 1(B)). Six months later, the macular edema was completely resolved (Figure 1(D)). One month before this ophthalmic visit, her glycemic control deteriorated to a glycated hemoglobin A1c of 7.4%, and her general physician prescribed 25 mg of oral empagliflozin, an SGLT2 inhibitor. The CRT in her left eye decreased to 253 *μ*m (Figure 1(D)) at one month after beginning the SGLT2 inhibitor treatment. Six months later (Figure 1(F)), her body weight was reduced, and the glycemic control was improved with the glycated hemoglobin A1c of 6.5% (Table 1). The CRT reduction was maintained for over two years without other ophthalmic therapy. The clinical course of the macular edema before and after the administration of the SGLT2 inhibitor is shown in Figure 1. The final decimal BCVA improved to 0.8, and the CRT measured by optical coherence tomography (OCT) was 235 *μ*m (Figure 1(H)) at 30 months after commencing the SGLT2 inhibitor.

### 2.2. Case 2

**Figure 2:**
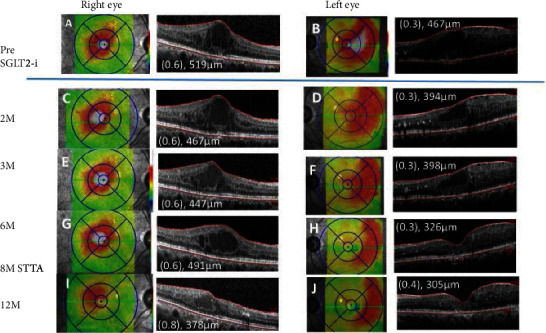
Case 2. Optical coherence tomographic images and fundus photographs showing the central retinal thickness (CRT) pre (A, B) and at 2 months (M) (C, D), 3 M (E, F), 6 M (G, H), and 12 M (I, J) after commencing the oral sodium-glucose cotransporter 2 inhibitor (SGLT2i). A sub-Tenon injection of triamcinolone acetonide (STTA) was administered in her right eye at eight months.

A 68-year-old woman was diagnosed with preproliferative DR 6 years earlier, and panretinal photocoagulation had been performed. However, the DME gradually developed beginning 5 years earlier. She was treated twice with intravitreal injections of ranibizumab and a sub-Tenon injection of triamcinolone acetonide in her left eye. In her right eye, she received 7 intravitreal injections of aflibercept and focal-grid laser photocoagulation. However, the DME persisted, and her decimal BCVA was 0.6 in the right eye and 0.3 in the left eye. The CRT was 519 *μ*m in the right eye and 467 *μ*m in the left eye (Figure 2(A) and (B)). Oral dapagliflozin was administered to improve the glycemic control. At the beginning of the dapagliflozin, her glycated hemoglobin A1c was 7.0%; however, her glycemic control was exacerbated by overconsumption; although, the refractory DME was improved. Two months later, the CRT was thinner in her left eye (Figure 2(D)), and six months later, the CRT was reduced to 491 *μ*m in the right eye (Figure 2(G)) and 326 *μ*m in the left eye (Figure 2(H)). Her BCVA was 0.6 in the right eye and 0.3 in the left. At six months, her body weight was reduced; although, the glycated hemoglobin A1c was elevated to 7.9% (Table 1). The clinical course of the macular edema is shown in Figure 2. A sub-Tenon injection of triamcinolone acetonide was administered to her right eye at eight months. Her final BCVA was 0.8 in the right eye and 0.4 in the left eye, and the CRT was 378 *μ*m in the right (Figure 2(I)), 305 *μ*m in the left (Figure 2(J)) at twelve months.

### 2.3. Case 3

**Figure 3:**
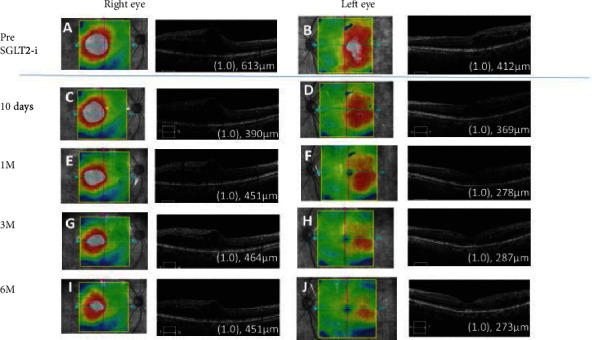
Case 3. Optical coherence tomographic images and fundus photographs showing the central macular thickness (CRT) pre (A, B) and at 10 days (C, D), 1 month (M) (E, F), 3 M (G, H), and 6 M (I, J) after commencing the oral sodium-glucose cotransporter 2 inhibitor (SGLT2i).

A 61-year-old woman was diagnosed with moderate nonproliferative DR without DME 7 years earlier. She had undergone bilateral cataract surgery 5 years earlier, and her BCVA had recovered to 1.0, but there was a gradual development of macular edema. She received 2 sub-Tenon injections of triamcinolone acetonide, 5 intravitreal injections of aflibercept, and focal laser treatment in her left eye. She received 1 intravitreal injection of aflibercept and a focal laser treatment in her right eye. Despite these treatments, the CRT was still 613 *μ*m in the right eye (Figure 3(A)) and 412 *μ*m in the left eye (Figure 3(B)); although, the decimal BCVA was 1.0 in both eyes. Her BCVAs were maintained, and she did not agree to continue anti-VEGF therapy. Oral luseogliflozin 2.5 mg was administered, and the CRT of both eyes (Figure 3(C) and (D)) began to decrease by two weeks after commencing the luseogliflozin. This reduction continued for six months. Six months later, her CRT was reduced to 451 *μ*m in the right eye (Figure 3(I)) and 273 *μ*m in the left eye (Figure 3(J)). The BCVA was maintained at 1.0 in both eyes. Her body weight was reduced at six months, and the glycated hemoglobin A1c was reduced from 6.5% to 6.1% at six months (Table 1).

## 3. Discussion

The findings in these three cases suggested that SGLT2 inhibitors can improve the chronic DME which was resistant to standard ophthalmic therapies. Anti-VEGF drugs are the first-line therapy for center-involved DMEs [1, 2]; however, they are costly, and clinical patients receive approximately three injections per year in Japan [8]. Our three patients did not agree to the frequent anti-VEGF injections due to their high costs, and reducing the number of anti-VEGF injections can lead to an overall decrease in the efficacy as seen in randomized trials [1, 2]. DME pathology persisted in all three cases following the multimodal therapy including steroid injections and focal/grid laser photocoagulation in our cases. However, stabilization and recovery were observed soon after the oral administration of a SGLT2 inhibitor. Notably, the improvements were detected as soon as two weeks after the initiation of the SGLT2 inhibitor in Case 3, and the improvements were maintained for at least 2 years in Case 1.

There is no evidence that internal medications have positive effects on DME, but two studies have reported the effectiveness of SGLT2 inhibitors for the treatment of DME [9, 10]. One study reported on the efficacy of SGLT2 inhibitors on the vitrectomized eyes of five patients [9]. However, vitrectomy is not the standard or even the preferential treatment for DME, and anti-VEGF therapy or other standard treatments were omitted in that study. An additional study presented a case of a DME patient who was given ipragliflozin. The patient was not treated with any anti-VEGF before the SGLT2-inhibitor administration, and OCT showed no significant changes after 24 weeks of SGLT2-inhibitor administration [10].

The precise mechanism by which SGLT2 inhibitors resolve the DME was not determined. Weight loss was observed in all cases, and the rapid effects of the SGLT2 inhibitors may be related to its diuretic effects. In Case 2, however, the blood glycemic level did not improve, suggesting that a short period of glycemic control improvement by SGLT2 inhibitors may not have a direct effect on the DME.

Empagliflozin reduces the risk of major adverse cardiovascular events [5] and reduces the progression of kidney diseases [6]. SGLT2 inhibitors may improve DME via systemic fluid adjustments.

One in vitro study reported that phlorizin, an SGLT inhibitor, attenuated the high glucose-induced morphological and functional changes in cultured bovine retinal pericytes [11].The expression of SGLT1 and SGLT2 in the eye including the retina has been reported in several papers [7, 12]. SGLT2 inhibitors remove excess glucose from the retinal microcirculation and hence reduce glucotoxicity, oxidative stress, low-grade inflammation, and restore insulin signaling. By preventing continued glucose-induced vascular dysfunction, the progression of microangiopathy and especially DR is improved [13, 14]. The long-lasting effect of SGLT2 inhibitors might be related to the reduction of the glucose-induced vascular endothelial dysfunction.

It has been well established that anti-VEGF therapy is the mainstay treatment for DME, and oral SGLT2 inhibitors combined with anti-VEGF injections may be additive and a good option for refractory DME.

There are some limitations in this study. Only three cases with widely different histories were studied, and all cases were undertreated with anti-VEGF agents compared to the standardized regimens. Additionally, three different SGLT2 inhibitors were prescribed to the three patients; all were effective in reducing the degree of DME, but their mechanism of action has not been clearly determined.

Therefore, we are planning a multicenter, randomized, open-label trial to investigate the safety and efficacy of combination therapy of anti-VEGF drug injections and SGLT2 inhibitors in patients with type 2 diabetes and DME [15].

In conclusion, our results showed an improvement of chronic DME after the initiation of oral SGLT2 inhibitors. SGLT2 inhibitors might be a novel, noninvasive, and low-cost treatment for DME.

## Figures and Tables

**Table 1:** Summary of three cases of DME pre and 6 months (M) after the SGLT2-i administration.

	Case 1		Case 2		Case 3	
	Pre	6 M after SGLT2-i	Pre	6 M after SGLT2-i	Pre	6 M after SGLT2-i
Age (yrs)/sex	66/F		68/F		61/F	
Body height (cm)	158		156		153	
Body weight (kg)	49.1	45.2	43	41.1	46.1	45
Blood pressure (mmHg)	150/82	136/62	128/70	126/74	124/77	121/70
SGLT2 inhibitor	—	Empagliflozin 25 mg	—	Dapagliflozin 5 mg	—	Luseogliflozin 2.5 mg
Diabetes treatment	Vildagliptin 100 mg Glimepiride 1.5 mg	Vildagliptin 100 mg	Alogliptin 25 mg Acarbose 300 mg Metformin 500 mg	Acarbose 300 mg Metformin 500 mg	—	—
Ophthalmic treatment; OD	Focal laser	—	Focal/grid laser IVA ×7	—	Focal laser, IVA ×1	—
; OS	Focal laser	—	IVR ×2, STTA ×2	—	Focal laser ×3, IVA ×5, STTA ×2	—
Central retinal thickness (*μ*m) OD/OS	223/598	230/232	519/467	491/326	613/412	451/273
Visual acuity OD/OS	(1.0)/(0.3)	(1.2)/(0.6)	(0.3)/(0.3)	(0.6)/(0.3)	(1.0)/(1.0)	(1.0)/(1.0)
HgA1c (%)	7.4	6.5	7.0	7.9	6.5	6.1
Blood glucose (mg/dL)	116	118	118	129	113	100
Total protein (g/dL)	ND	6.5	6.4	6.9	7.6	ND
Albumin (g/dL)	ND	ND	ND	4.1	4.2	ND
Aspartate aminotransferase (U/L)	ND	18	12	12	21	22
Alanine aminotransferase (U/L)	ND	13	11	11	18	18
Creatinine (mg/dL)	0.47	0.42	0.61	0.65	0.59	0.62
U-protein (qualitative analysis)	—	—	—	±	—	—
U-albumin (mg/g・Cre)	8.7	6.9	ND	ND	24.8	ND

DME: diabetic macular edema; SGLT2-i: SGLT2 inhibitor; OD: oculus dexter; OS: oculus sinister; IVA: intravitreal injection of aflibercept; IVR: intravitreal injection of ranibizumab; STTA: sub-Tenon injection of triamcinolone acetonide.
